# Supplementary material for: Chromothripsis during telomere crisis is independent of NHEJ, and consistent with a replicative origin
Source: Genome Res. 2019 May;29(5):737–49. doi: 10.1101/gr.240705.118 (PMC6499312; doi:10.1101/gr.240705.118)
Supplement: Supplemental Material [file supp_gr.240705.118_Supplemental_file_1.zip › contigs/annotated_contigs/DB53/contig.2.DB53_length_469_mean_cov_8.18123667377.docx]

**DB53_length_469_mean_cov_8.18123667377**

```
CTGGGACTAAGGTGTGTGCCACCATACCTGGCTAATTTTTGTATTTTTTAGTAGAAATGGGGTTTCACCATATTGGCCTGGCTGGTCTC
 >chr3:64296642-64296865 + E=1e-119 p=3e-02
GAACTCCTGACCTCGTGATCCGTCTACCTTGGCCTCCCAAAGTGCTGGGATTACAGGCCTGAGCCACCGCACCCAACCTAAATAAATAT

TTTTAAAATTAGAGTTTGAATAAAAGATTCAAAGGAAAATGG|ACA|ACCACACTGCAAAGAAACCATTCTTGTCTCTATCTGAGATGA
                                           >chr3:64303858-64304107 + E=2e-138
GGAAACTGTTACGTGGTTGAAATGGAATTTGACCACAGGGTCCTGTAGTACAAAAGCACACTCAATTAACTGCTAGGTCATCTTCTAAC

TCCAATCCCTTGTTATCTAGGTCTTCAAAGCAAGAACTTCTATTGTACCATTGTACCTACTATATGCCAGGAACTGTTCTAGGCATTGA

TGATAAAGCAGTGATCAAAACAAGCA
```
